# An ancestral stomatal patterning module revealed in the non-vascular land plant *Physcomitrella patens*

**DOI:** 10.1242/dev.135038

**Published:** 2016-09-15

**Authors:** Robert S. Caine, Caspar C. Chater, Yasuko Kamisugi, Andrew C. Cuming, David J. Beerling, Julie E. Gray, Andrew J. Fleming

**Affiliations:** 1 Department of Animal and Plant Sciences, University of Sheffield, Sheffield S10 2TN, UK; 2 Department of Molecular Biology and Biotechnology, University of Sheffield, Sheffield S10 2TN, UK; 3 Centre for Plant Science, University of Leeds, Leeds LS2 9JT, UK

**Keywords:** Stomata, Evolution, Patterning, Peptide signalling

## Abstract

The patterning of stomata plays a vital role in plant development and has emerged as a paradigm for the role of peptide signals in the spatial control of cellular differentiation. Research in *Arabidopsis* has identified a series of epidermal patterning factors (EPFs), which interact with an array of membrane-localised receptors and associated proteins (encoded by ERECTA and TMM genes) to control stomatal density and distribution. However, although it is well-established that stomata arose very early in the evolution of land plants, until now it has been unclear whether the established angiosperm stomatal patterning system represented by the EPF/TMM/ERECTA module reflects a conserved, universal mechanism in the plant kingdom. Here, we use molecular genetics to show that the moss *Physcomitrella patens* has conserved homologues of angiosperm EPF, TMM and at least one ERECTA gene that function together to permit the correct patterning of stomata and that, moreover, elements of the module retain function when transferred to *Arabidopsis*. Our data characterise the stomatal patterning system in an evolutionarily distinct branch of plants and support the hypothesis that the EPF/TMM/ERECTA module represents an ancient patterning system.

## Abstract (precis)

**Summary:** The genetic module controlling patterning of stomata in vascular plants also functions in non-vascular plants, consistent with the idea that it represents an ancestral mechanism in plant evolution.

## INTRODUCTION

Stomata are microscopic pores present in the epidermis of all angiosperms and the majority of ferns and bryophytes. Evolution of stomata has proved to be an essential step in the success and diversification of land plants over the past 400 million years (Beerling, 2007). In particular, this innovation, coupled with vascular tissues and a rooting system, enabled land plants to maintain hydration by regulating the plant-soil-atmosphere water flows under fluctuating environmental conditions (Berry et al., 2010; Raven, 2002; Vatén and Bergmann, 2012). Stomatal distribution is tightly regulated, both by endogenous developmental mechanisms that influence their number and pattern in different organs of the plant, and by modulation of these controls by a host of environmental factors (Chater et al., 2015; Geisler et al., 1998; Hunt and Gray, 2009; MacAlister et al., 2007). This spatial control of stomatal distribution, combined with the ease of scoring phenotype on the exposed epidermis, makes them an attractive system to investigate the control of patterning in plants, a major topic highlighted in the seminal work by Steeves and Sussex (1989).

Extensive molecular genetic analyses in the model flowering plant *Arabidopsis* have provided significant insight into the mechanisms controlling stomatal patterning and differentiation in angiosperms (Chater et al., 2015; Engineer et al., 2014; Pillitteri and Torii, 2012; Simmons and Bergmann, 2016). In *Arabidopsis*, negatively and positively acting secreted peptide signals [epidermal patterning factors (EPFs) and epidermal patterning factor-like proteins (EPFLs)] function to control where and when stomata form and ensure that stomata are separated from each other by at least one intervening epidermal cell, thus optimising leaf gas exchange (Abrash and Bergmann, 2010; Hara et al., 2007, 2009; Hunt et al., 2010; Hunt and Gray, 2009; Sugano et al., 2010). This ‘one cell spacing rule’ results from the stereotypical local pattern of cell divisions by which stomata form, accompanied by cross-talk between cells. The molecular mechanism enforcing the spacing rule involves EPF(L)s interacting with transmembrane receptors, including those encoded by members of the ERECTA gene family (*ERECTA*, *ER*; *ERECTA-LIKE1*, *ERL1*; and *ERECTA-LIKE2*, *ERL2*) activity of which is modulated in stomatal precursor cells by the receptor-like protein TOO MANY MOUTHS (TMM) (Lee et al., 2015, 2012; Shpak et al., 2005; Torii, 2012). Binding of EPF(L)s entrains a well-characterised signal transduction pathway involving a series of mitogen-activated protein kinases, which leads to the cellular events of stomatal differentiation (Torii, 2015).

Little is known of the developmental mechanisms regulating stomatal patterning in early land plants. Fossil cuticles of 400-million-year-old small branching leafless vascular land plants, such as *Cooksonia*, indicate stomata were generally scattered more or less evenly across stem surfaces without clustering (Edwards et al., 1998) and these authors report that in the Rhynie Chert fossil plants stomata commonly occur on ‘an expanded portion of the axis just below the sporangium’. These observations suggest the existence of a stomatal patterning module early in land plant evolution but we have very limited information on the nature of the genetic module controlling this process. However, homologues of key genes regulating vascular land plant stomatal differentiation are present in the genome and are expressed during sporophyte development in the moss *Physcomitrella patens* (Chater et al., 2013; O'Donoghue et al., 2013; Ortiz-Ramírez et al., 2016; Vatén and Bergmann, 2012), a basal non-vascular land plant lineage with stomata. This suggests that genetic components involved in regulating stomatal spacing have been conserved between mosses and vascular plants. This notion is further supported by complementation work performed in *Arabidopsis* showing that *Physcomitrella patens* group 1A basic helix-loop-helix transcription factors can at least partially fulfil the function of their angiosperm counterparts in the regulation of stomatal development (MacAlister and Bergmann, 2011).

Here, we use molecular genetics to compare stomatal patterning systems in a bryophyte (*Physcomitrella patens*) and an angiosperm (*Arabidopsis thaliana*). We show that *P. patens* has an EPF/TMM/ERECTA module required for stomatal patterning fundamentally similar to that found in angiosperms and that elements of the module retain function when transferred to *Arabidopsis*. Our data characterise the stomatal patterning system in moss and are consistent with the hypothesis that the EPF/TMM/ERECTA module represents an ancient patterning system in plants.

## RESULTS

**Fig. 1.**
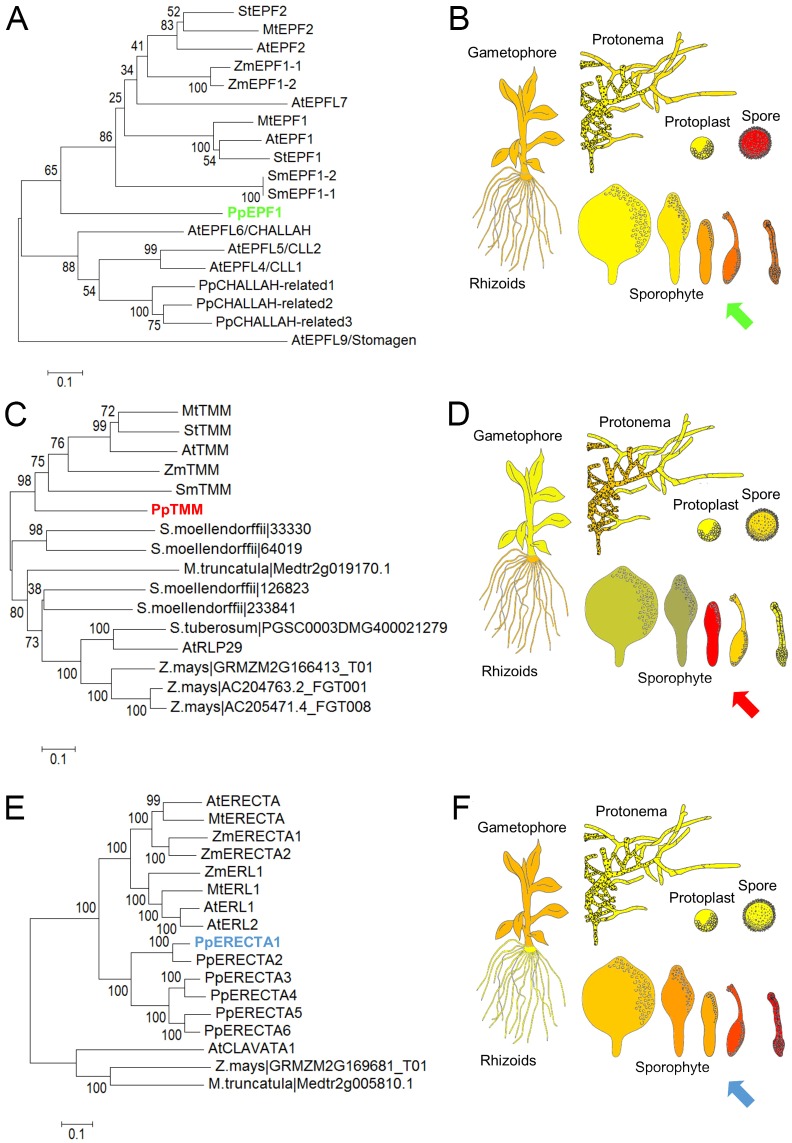
**Phylogeny and expression profiles of stomatal patterning genes in *Physcomitrella patens*.** (A,C,E) Phylogenetic trees constructed using amino acid sequences of selected *Arabidopsis EPF1* (A), *TMM* (C) and ERECTA (E) gene family members based on Phytozome V11 (Goodstein et al., 2012), using the neighbour-joining method (Saitou and Nei, 1987; Takata et al., 2013) on MEGA6 (Tamura et al., 2013). The percentage of replicate trees in which the associated taxa clustered together in the bootstrap test (1000 replicates) are shown next to the branches (Felsenstein, 1985). Amino acid sequences from *P. patens* (Pp), *Selaginella* *moellendorffii* (Sm), *Zea* *mays* (Zm), *Symphytum* *tuberosum* (St)*,* *Medicago* *truncatula* (Mt) and *A. thaliana* (At) were used to generate trees, except for ERECTA, for which *S. moellendorffii* and *S. tuberosum* gene family members were omitted, owing to the large overall number of genes in the ERECTA family. For complete analyses of all three gene families, see Fig. S1. (B,D,F) Expression profiles of *PpEPF1* (B), *PpTMM* (D) and *PpERECTA1* (F) based on microarray data taken from the *P. patens* eFP browser (Ortiz-Ramírez et al., 2016; Winter et al., 2007) for spore, protoplast, protonemal, gametophyte and sporophyte tissue. Red indicates a relatively high transcript level, with the arrows highlighting phases of sporophyte development when the respective genes appear to be relatively highly expressed. For the expression profiles of other PpERECTA gene family members, see Fig. S2.

To identify potential orthologues of angiosperm genes implemented in stomatal patterning in *P. patens*, we performed a bioinformatic analysis. As shown in Fig. 1A and Fig. S1A, a single homologue of *Arabidopsis EPF1* and *EPF2* exists in *P. patens*, *PpEPF1* (see also Takata et al., 2013)*.* Similarly, the stomatal patterning protein TMM (which is encoded by a single gene in *Arabidopsis*) is homologous to a single gene in *P. patens*, termed *PpTMM* (Peterson et al., 2010) (Fig. 1C; Fig. S1B). The situation with the ERECTA genes is more complicated as six potential orthologues are found in the genome of *P. patens* (Villagarcia et al., 2012) (Fig. 1E; Fig. S1C).

To identify genes potentially involved in stomatal patterning, we first interrogated a microarray database (O'Donoghue et al., 2013) to ascertain which PpERECTA genes showed upregulation of expression in the developing sporophyte. All the PpERECTA genes were expressed to some level in the sporophyte but only *PpERECTA1* was upregulated relative to protonemal tissue (Fig. S2A), and qRT-PCR analysis confirmed that *PpERECTA1* expression was significantly upregulated in the sporophyte (Fig. S2B). This was further indicated by the analysis of two other transcriptomic data sets accessible via phytozome V11 and the eFP browser at bar.utoronto.ca (see Table S1 for accession numbers), which showed a relatively high level of *PpERECTA1* expression in the sporophyte (Fig. S2C,D) (Goodstein et al., 2012; Ortiz-Ramírez et al., 2016; Winter et al., 2007). Taken together, the data suggested that *PpERECTA1* expression was increased in the sporophyte and, thus, might be involved in stomatal patterning. As shown in Fig. 1B,D,F, analysis of eFP Browser data for *PpEPF1*, *PpTMM* and *PpERECTA1* indicated an accumulation of the relevant transcripts in young sporophyte tissue.

**Fig. 2.**
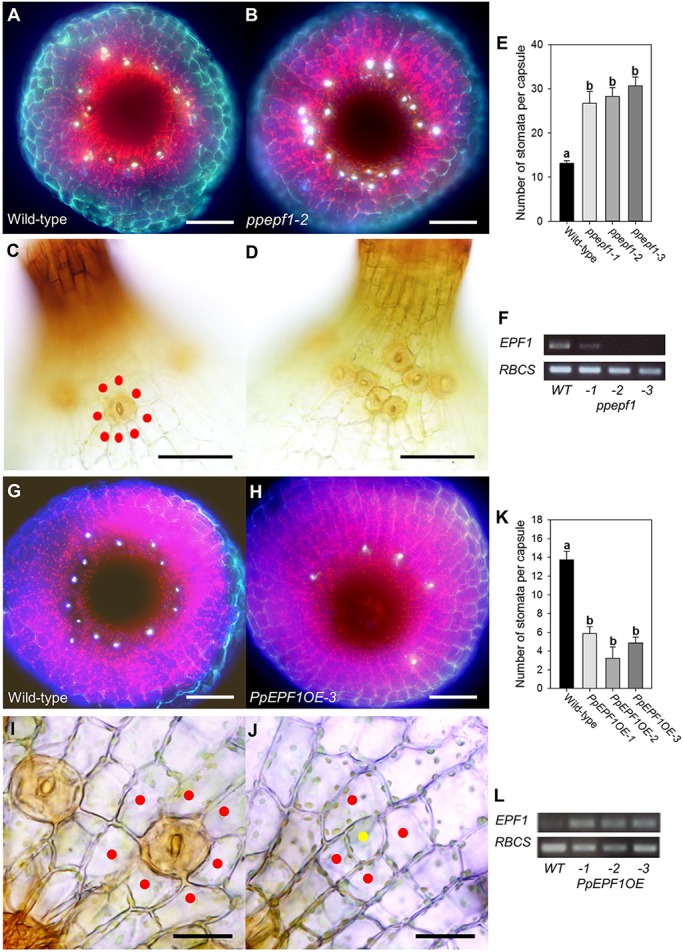
**EPF function is conserved in *Physcomitrella patens*.** (A,B) Fluorescence images of the base of the sporophyte from WT (A) and *ppepf1-2* (B) plants. Stomata (bright white fluorescence) are spaced around the base in a ring with an increased number in *ppepf1-2*. (C,D) Bright-field lateral views of the sporophyte base from WT (C) and *ppepf1-2* (D) plants. In WT, stomata are surrounded by epidermal cells (red dots) whereas in *ppepf1-2* stomata occur in clusters. (E) Number of stomata per capsule in WT and three *ppepf1* mutant lines. Lines indicated with different letters can be distinguished from each other (*P*<0.001; one-way ANOVA with multiple comparisons corrected using a Dunnett's test; *n*=7). (F) RT-PCR analysis of the WT and transgenic lines shown in E with expression of *PpEPF* (upper panel) and a *PpRBCS* control (lower panel). (G,H) Fluorescence images of the base of the sporophyte from WT (G) and *PpEPF1OE* (H) plants. Fewer stomata are visible in the *PpEPF1OE* sporophyte. (I,J) Bright-field lateral views of the sporophyte base from WT (I) and *PpEPF1OE* (J) plants with a possible stomatal precursor (yellow dot) indicated. (K) Number of stomata per capsule in WT and three *PpEPFOE* mutant lines. Lines indicated with different letters can be distinguished from each other (*P*<0.001; one-way ANOVA with multiple comparisons corrected using a Dunnett's test; *n*=8). (L) RT-PCR analysis of the WT and transgenic lines shown in K with expression of *PpEPF1* (upper panel) and *PpRBCS* control (lower panel) transcripts. Error bars indicate s.e.m. Scale bars: 100 µm (A,B,G,H); 50 µm (C,D); 25 µm (I,J).

Having identified genes encoding homologues for each of the components of the core EPF/TMM/ERECTA module involved in angiosperm stomatal patterning, we undertook a functional analysis in *P. patens* by creating a series of gene knockouts and analysing stomatal patterning in the sporophytes of the transgenic plants. Interruption of the targeted locus in transgenic plants was confirmed by genomic PCR (Fig. S3). As shown in Fig. 2A-F, loss of *PpEPF1* function led to an increase in the number of stomata per capsule. The extra stomata formed at the appropriate location at the base of the sporophyte (Fig. 2A,B), i.e. they did not extend ectopically into the flanks of the spore capsule. As a consequence, stomata in *ppepf1* knockout mutant capsules frequently occurred in clusters that were not apparent in wild-type (WT) sporophytes, where most stomata are separated from each other by at least one neighbouring epidermal cell (Fig. 2C,D). Quantification confirmed an increased number of stomata per capsule in the sporophytes of three independently generated *ppepf1* knockout lines (Fig. 2E). Expression analysis confirmed the absence (lines *ppepf1-2*, *ppepf1-3*) or greatly decreased transcript level (*ppepf1-1*) for *PpEPF1* in these plants (Fig. 2F). Interruption of the targeted locus in transgenic plants was verified by genomic PCR (Fig. S3).

We also characterised the outcome of increased expression of *PpEPF1* on stomatal formation by creating lines of transgenic *P. patens* in which the *PpEPF1* coding sequence was constitutively overexpressed via the rice actin promoter (Fig. 2L). Sporophytes of the transgenic plants displayed a phenotype with a greatly reduced number of stomata. At the base of the sporophyte stomata were sporadic (Fig. 2G,H) and the number of stomata per capsule significantly decreased in three independent lines overexpressing *PpEPF1* (Fig. 2K). Although the number of mature stomata was clearly decreased in the plants overexpressing *PpEPF1*, analysis of the epidermis at the base of the sporophytes of the transgenic plants indicated occasional division patterns suggestive of the formation of stomatal precursors that had failed to undergo further differentiation into the stomatal lineage (compare Fig. 2I and 2J).

**Fig. 3.**
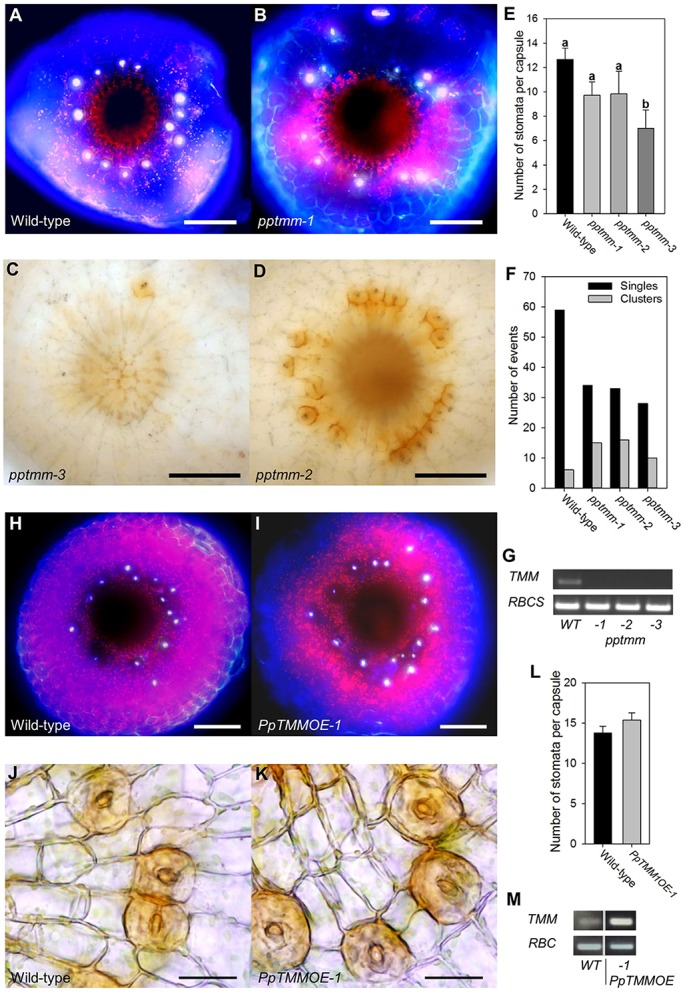
**TMM functions in stomatal patterning in *Physcomitrella patens*.** (A,B) Fluorescence images of the base of the sporophyte from WT (A) and *pptmm-1* (B) plants. The pattern of stomata (bright white fluorescence) is disrupted in the *pptmm-1* mutant. (C,D) Bright-field lateral views of the sporophyte base from two transgenic lines: *pptmm-3* (C) and *pptmm-2* (D). The number and patterning of stomata varies from plant to plant in each of the three independent *pptmm* lines. (E) Number of stomata per capsule in WT and three *pptmm* mutant lines. Lines indicated with different letters can be distinguished from each other (*P*<0.05; one-way ANOVA with multiple comparisons corrected using a Dunnett's test; *n*>6). (F) Percentage of stomata in clusters in the lines shown in E. (G) RT-PCR analysis of the WT and transgenic lines shown in E with expression of *PpTMM* (upper panel) and *PpRBCS* control (lower panel) transcripts. (H,I) Fluorescence images of the base of the sporophyte of WT (H) and *PpTMMOE* (I) plants. (J,K) Bright-field lateral views of the sporophyte base from WT (J) and *PpTMMOE* (K) plants. (L) Number of stomata per capsule in WT and *PpTMMOE* line. No significant difference (*P*<0.05) was found between the lines (one-way ANOVA with multiple comparisons corrected using a Dunnett's test; *n*=8). (M) RT-PCR analysis of the WT and transgenic line shown in L with expression of *PpTMM* (upper panel) and *PpRBCS* control (lower panel) transcripts. Error bars indicate s.e.m. Scale bars: 100 µm (A,B,H,I); 50 µm (C,D); 25 µm (J,K).

To investigate the role of the PpTMM receptor, we generated independent knockout lines. Examples of the range of phenotypes observed are shown in Fig. 3B-D for comparison with the WT pattern (shown in Fig. 3A). Some capsules had exceptionally few stomata (Fig. 3C) whereas others developed numerous stomata, many of which occurred in clusters (Fig. 3D). This variation was consistently observed across all three independent *pptmm* knockout lines. Again, as with the *ppepf1* knockout and WT lines, stomata formation remained restricted to the base of the capsule. Quantification of the transgenic sporophytes revealed that the number of stomata per capsule tended to be lower in the *pptmm* knockout lines than in the WT control, although this was statistically significant only in the line *pptmm-3* (Fig. 3E)*.* When the proportion of stomata forming in clusters (defined as stomata forming in pairs or higher order adjacent complexes) was measured, it was apparent that the *pptmm* knockout lines had a higher number of stomata in clusters than WT (Fig. 3F). Interruption of the targeted locus in transgenic plants was verified by genomic PCR (Fig. S3) and expression analysis confirmed that the three *pptmm* knockout lines contained no detectable *PpTMM* transcript (Fig. 3G).

We further investigated the role of *PpTMM* by analysing transgenic *P. patens* in which the *PpTMM* sequence was constitutively overexpressed (Fig. 3M). For this part of the investigation we were only able to identify a single transgenic line but analysis suggested that an increased level of *PpTMM* transcripts had little effect on stomatal patterning. There was a slight increase in the number of stomata per capsule (Fig. 3H,I) but quantification indicated that this was not statistically significant (Fig. 3L). The extent of stomatal clustering was similar to that observed in WT sporophytes (Fig. 3J,K).

**Fig. 4.**
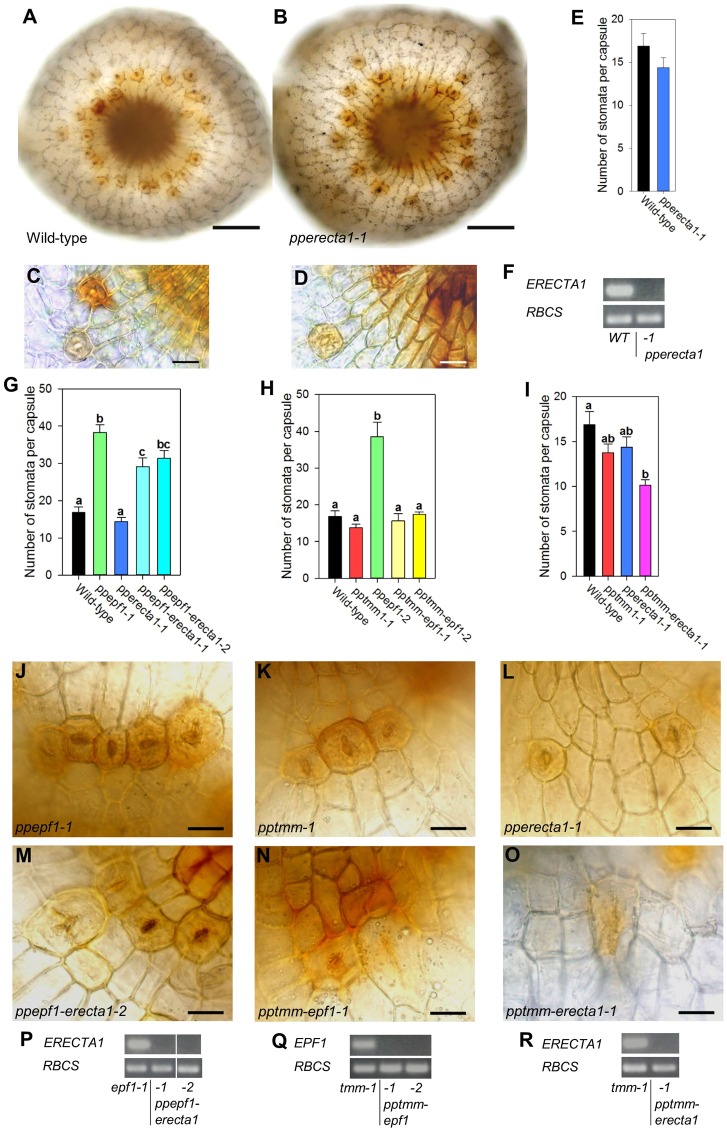
**Epistasis between *PpEPF1*, *PpTMM* and *PpERECTA1* supports a concerted role in stomatal patterning.** (A,B) Bright-field images of the base of the sporophyte from WT (A) and *pperecta1-1* (B) plants. (C,D) Bright-field lateral views of the sporophyte base from WT (C) and *pperecta1-1* (D) plants. (E) Number of stomata per capsule in WT and a *pperecta1-1* mutant line. No significant difference (*P*<0.05) was found between the lines (one-way ANOVA with multiple comparisons corrected using a Dunnett's test; *n*=8). (F) RT-PCR analysis of WT and *pperecta1-1* with expression of *PpERECTA1* (upper panel) and *PpRBCS* control (lower panel) transcripts. (G-I) Number of stomata per capsule in *ppepf1*-*erecta1* (G), *pptmm*-*epf1* (H) and *pptmm*-*pperecta1* (I) double mutants. Within each panel, lines indicated with different letters can be distinguished from each other (*P*<0.05; one-way ANOVA with multiple comparisons corrected using a Tukey test; *n*=8). (J-O) Bright-field lateral views of the base of sporophytes from *ppepf1* (J), *pptmm* (K), *pperecta1* (L), *ppepf1-erecta1-2* (M), *pptmm-epf1-1* (N) and *pptmm-erecta1-1* (O) lines. (P-R) RT-PCR analysis of the mutant lines shown in M-O with the upper panel showing the transcript detection for *PpERECTA1* (P,R) or *PpEPF1* (Q), as indicated. Lower panel in each case indicates transcript detection for a *PpRBCS* control. Error bars in E,G-I indicate s.e.m. Scale bars: 100 µm (A,B); 25 µm (C,D,J-O).

To ascertain whether *P. patens* requires ERECTA gene functioning during stomatal development, we next targeted the *PpERECTA1* gene. Only a single *PpERECTA1* knockout line was identified and, as shown in Fig. 4A,B, stomata formed in the appropriate position at the base of the sporophyte with no obvious difference in stomatal differentiation (Fig. 4C,D) and no effect on stomatal number per capsule (Fig. 4E). Loss of *PpERECTA1* gene expression in this line was confirmed by RT-PCR (Fig. 4F), as was interruption of the targeted locus by genomic PCR (Fig. S3). Because analysis of the *pperecta1* knockout was unable to establish a conclusive role for this component in stomatal development, further experiments were carried out. To understand whether the *PpTMM* and *PpEPF1* genes were acting in the same pathway as *PpERECTA1* during stomatal development, a series of double knockout mutants were produced. Analysis of *ppepf1*-*erecta1* double knockouts indicated a diminished *ppepf1* phenotype. Thus, although more stomata per capsule developed compared with WT the increase was less than in the *ppepf1* mutant (Fig. 4G). An even more dramatic effect was observed when *pptmm-epf1* double knockouts were generated. In this situation, the phenotype of increased stomata per capsule observed in the *ppepf1* knockout was found to be entirely dependent on the presence of a functional *PpTMM* gene (Fig. 4H). Finally, a *pptmm*-*pperecta1* double knockout displayed a greater decrease in stomata per capsule than observed in the single *pptmm* and *pperecta1* mutants (Fig. 4I). Analysis of epidermal regions of capsules from the different knockout combinations (Fig. 4J-O) suggested that, in addition to the differences in stomata number, loss of some EPF/TMM/ERECTA gene combinations influenced the positioning/form of stomata and the general pattern of cell division in the epidermis. For example, although loss of *PpERECTA1* in a *ppepf1* background led to a decrease in stomatal number per capsule, there was often an apparent disruption to the epidermal cell patterning in the vicinity of the stomata that were formed (Fig. 4J,M). In the case of the *ppepf1*-*pptmm* double knockouts (which restored stomata number per capsule to wild-type levels), there were also alterations to the epidermal cell division planes from the patterns observed in the *ppepf1* or *pptmm* capsules (Fig. 4J,K,N). Furthermore, guard cells that formed at the boundaries appeared stretched, taking on a shape akin to neighbouring epidermal pavement cells. This elongated guard cell conformation was also seen in the *pptmm*-*pperecta1* line (Fig. 4L,O). Analysis of the various double knockout combinations described above confirmed the absence of the relevant transcripts (Fig. 4P-R) and interruption of the targeted loci (Fig. S3).

In addition to suppression of stomata formation, some EPF-like peptides in angiosperms have evolved to inhibit EPF action competitively (Lee et al., 2015; Ohki et al., 2011). Most notably, *AtEPFL9* (*STOMAGEN*) has been shown to enhance stomata formation in *Arabidopsis* and overexpression of *STOMAGEN* leads to increased stomatal number in *Arabidopsis* (Hunt et al., 2010; Sugano et al., 2010). As indicated in Fig. 1 and Fig. S2A, bioinformatic analysis indicates that the *P. patens* genome encodes a peptide similar to EPF2 (which in *Arabidopsis* inhibits stomatal development), but has no apparent equivalent to STOMAGEN (which in *Arabidopsis* antagonises the activity of EPF2 and stimulates stomatal development). To investigate whether the stomatal patterning system in *P. patens* could be disrupted by overexpression of the evolutionarily distinct antagonistic peptide STOMAGEN (Fig. S4A), we overexpressed the *Arabidopsis STOMAGEN* gene in a WT *P. patens* background. Our results indicated that although the *STOMAGEN* transcripts accumulated to a high level (Fig. S4G), there was no apparent phenotype in terms of altered numbers of stomata per capsule (Fig. S4B-D) although abnormal epidermal cells and aberrant guard cells were occasionally observed at the base of the transgenic capsules (Fig. S4E,F).

Because our data suggested that *PpEPF1*, *PpTMM* and *PpERECTA1* all play a role in stomatal patterning in *P. patens*, we investigated whether they might represent conserved functions by introducing the *P. patens* genes into the appropriate *Arabidopsis* genetic background, i.e. could they complement the cognate angiosperm gene function in stomatal patterning? For this experiment, we focused on the putative EPF and TMM orthologues because the respective mutants in *Arabidopsis* have clear phenotypes with respect to stomatal density and patterning. In leaves, loss of *AtEPF1* or *AtEPF2* results in increased stomatal density, with stomatal clustering being especially pronounced in *atepf1* (Hara et al., 2007). In *atepf2*, increased density is the result of increased entry of cells to the stomatal lineage, which causes not only more stomata but also more small epidermal stomatal precursor cells (Hara et al., 2009; Hunt and Gray, 2009). In *Arabidopsis tmm*, stomatal phenotype varies depending on the organ. For example, in leaves stomatal density and clustering is markedly increased, in *tmm* inflorescence stems no stomata are found, and in the flower pedicel a gradient of stomatal density is observed. Thus, at the base of *tmm* pedicels there are no stomata, in the middle region a few stomata form, and at the apical region of the pedicel stomatal density exceeds that of wild type and clustering is common (Bhave et al., 2009; Geisler et al., 1998; Yang and Sack, 1995).

**Fig. 5.**
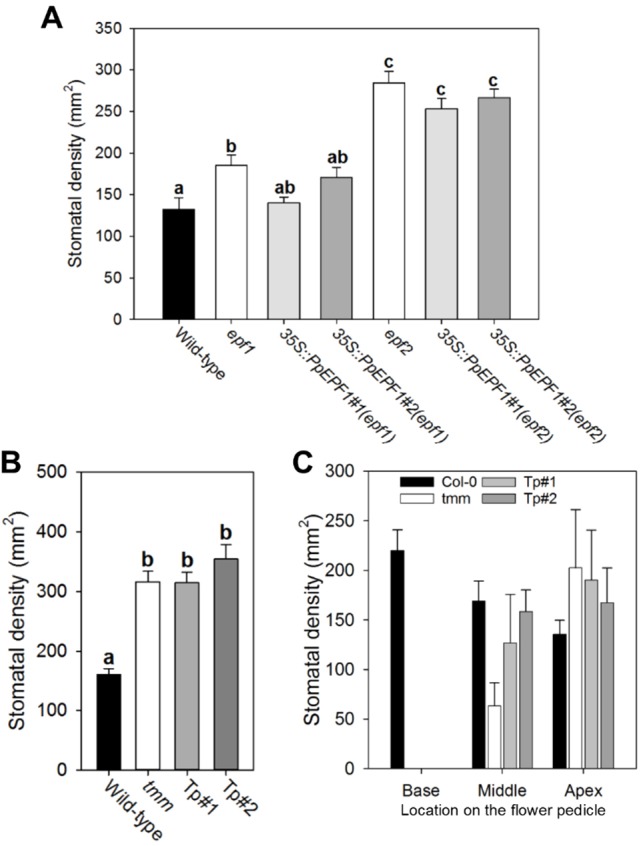
***PpEPF1* and *PpTMM* can partially rescue *Arabidopsis* stomatal density phenotypes.** (A) Stomatal density in leaves in a series of lines of *Arabidopsis thaliana* either lacking *AtEPF1* (*epf1*) or *AtEPF2* (*epf2*) and overexpressing the *PpEPF1* sequence (35S::*PpEPF1*). Stomatal density in WT leaves is shown as a control. (B) As in A but for two lines of the *Arabidopsis tmm* mutant complemented with the *PpTMM* sequence under control of the native *AtTMM* promoter (Tp). In A and B, lines indicated with different letters can be distinguished from each other [*P*<0.05; one-way ANOVA with multiple comparisons corrected using a Tukey test; *n*=6 (A), *n*=8 (B)]. (C) As in B but for the phenotype observed in base, middle or apex of the flower pedicel (as indicated). Error bars=s.e.m.

When *PpEPF1* was constitutively overexpressed in *atepf1* we found that the mutant phenotype was partially rescued, with leaves having stomatal densities that were lower than in the *atepf1* background and which approached wild-type values (Fig. 5A). When *PpEPF1* was overexpressed in the *atepf2* mutant background only a slight recovery of stomatal density occurred (Fig. 5A) and epidermal cell density was essentially unchanged relative to that observed in *atepf2* (Fig. S5). With respect to *PpTMM*, when this sequence was expressed in the *Arabidopsis attmm* mutant under control of an endogenous *AtTMM* promoter there was no overt restoration of stomatal density to wild-type values in leaves and stomatal clustering was still observed (Fig. 5B). However, when the pedicel was examined there was a partial rescue of the *attmm* phenotype. This was most obvious in the middle region where stomatal density was restored towards wild-type values whereas in the basal and apical regions stomatal densities were similar to those observed in the *tmm* mutant (Fig. 5C).

## DISCUSSION

The control of patterning is core to development and the EPF/TMM/ERECTA module has emerged as a paradigm for peptide signalling in plants to control the distribution of essential cellular complexes on the epidermis, the stomata. Although it is well-established that stomata arose very early in the evolution of land plants, until now it has been unclear whether the angiosperm stomatal patterning system represents an ancient, universal mechanism in the plant kingdom. Our data indicate that an essentially similar system functions in the moss *P. patens*, providing strong evidence that the EPF/TMM/ERECTA module represents an ancestral patterning system for stomata. Mosses and flowering plants last shared a common ancestor over 400 million years ago (Ruszala et al., 2011), suggesting that the leafless sporophytes of early vascular land plants may have deployed a patterning module comprising genes closely related to the EPF/TMM/ERECTA suite identified here.

Our data establish, firstly, that the genome of an extant bryophyte, *P. patens*, contains sequences homologous to all three components of the EPF/TMM/ERECTA module present in the angiosperm *A. thaliana* and that they are expressed at an appropriate time in development to play a role in stomatal patterning. Two of these components (*PpEPF1* and *PpTMM*) are present as single-copy genes, consistent with them representing relatively ancient ancestral forms. The situation with the ERECTA gene family was more complicated, but our expression analysis, including the analysis of staged, dissected sporophyte tissue, allowed us to identify one member of the ERECTA family in *P. patens* (*PpERECTA1*) that was expressed at the appropriate time and place to play a role in stomata formation and which was therefore selected for further investigation (O'Donoghue et al., 2013).

Functional analysis of these three genes (*PpEPF1*, *PpTMM*, *PpERECTA1*) indicated that they are all involved in stomatal patterning in *P. patens* with roles not dissimilar to those played by their putative orthologues in *Arabidopsis* (Geisler et al., 1998; Hara et al., 2007, 2009; Hunt and Gray, 2009; Shpak et al., 2005; Yang and Sack, 1995). This was clearest with *PpEPF1*. Loss of this peptide led to an increase in stomatal clustering and in number of stomata per capsule whereas overexpression led to a decrease in stomata per capsule. This indicates a function directly comparable to that observed for *AtEPF1* and, to a lesser extent, *AtEPF2* in *Arabidopsis* where loss of function leads to an increase in leaf stomatal density and stomatal clustering (Hara et al., 2007, 2009; Hunt and Gray, 2009).

With respect to *PpTMM*, a more complicated picture emerged, consistent with the context-dependence of the *tmm* phenotype reported in *Arabidopsis* (Geisler et al., 1998; Yang and Sack, 1995). For example, in mature leaves of the *Arabidopsis attmm* mutant stomatal clustering is apparent and stomatal density is higher than that of WT, whereas at the base of the pedicels no stomata form, in the middle region some stomatal formation occurs (with some clustering), and at the top of the pedicel ectopic stomata form, leading to increased density and clustering relative to the WT (Bhave et al., 2009; Geisler et al., 1998). In the sporophyte of *P. patens* (where stomata only form at the base of the spore capsule), we observed an overall trend for a decrease in stomatal density and increase in clustering in the *pptmm* lines. However, these average values obscure significant spatial variation even within single spore capsules, so that on a given capsule it was not uncommon to observe both stomatal clustering and adjacent areas devoid of stomata, i.e. the phenotype encompassed elements observed on leaves and pedicels in *Arabidopsis*. The mechanistic basis of this variation awaits elucidation but the data indicate an overall conservation of sequence and function for TMM in *P. patens* and *Arabidopsis* and support an important ancestral role for this protein in the modulation of stomatal patterning in leafless early land plants. It is possible that the regulation of stomatal stochasticity by TMM in early land plants enabled or facilitated the later evolution of distinct stomatal patterns in different parts of the plant. One possibility is that other peptides in *P. patens*, encoded by genes similar to *Arabidopsis EPFL6* (*CHALLAH*), *EPFL5* (*CHALLAH-LIKE 1*) and *EPFL4* (*CHALLAH-LIKE 2*), play a role in inhibiting stomatal formation in the absence of *PpTMM*, as is the case in *Arabidopsis* (Abrash and Bergmann, 2010; Abrash et al., 2011). A recent bioinformatics study has identified nine PpEPFL (CHALLAH-like) genes that are upregulated in the developing sporophyte (Ortiz-Ramírez et al., 2016; Takata et al., 2013). These genes represent a target for future work to provide a deeper understanding of peptide signalling and stomatal patterning.

In *Arabidopsis*, TMM modulates the activity of ERECTA proteins and the action of AtEPF2 (and possibly AtEPF1) is dependent on TMM (Hara et al., 2007; Hunt and Gray, 2009; Lee et al., 2012). To test whether PpEPF1 action requires PpTMM, we produced double mutant lines (*pptmm-epf1*) and found that the *ppepf1* phenotype was masked. Thus, there is an epistatic interaction between *PpTMM* and *PpEPF1* similar to the situation reported in *Arabidopsis* for EPF1 or EPF2 and TMM (Hara et al., 2007, 2009; Hunt and Gray, 2009).

Our analysis of *P. patens* sporophytes lacking *PpERECTA1* expression indicated no difference in stomatal number and only a slight difference in the spacing of stomata. As only one knockout line could be assessed we emphasise that this result should be interpreted with caution. However, analysis of *pperecta1* in combination with either *ppepf1* or *pptmm* indicated a more pronounced role for PpERECTA1 in stomatal patterning. For example, loss of *PpERECTA1* partially rescued the phenotype shown by the *ppepf1* knockout mutant. The available data indicate that there are at least five other closely related PpERECTA genes expressed in the sporophyte (Fig. 2C; Fig. S1C) (Villagarcia et al., 2012), so the lack of phenotype in the single *PpERECTA1* knockout mutant may reflect a degree of genetic redundancy in a manner similar to the redundant activity of this receptor family in *Arabidopsis* (Shpak et al., 2005). Further analysis of these PpERECTA genes in the context of *pptmm* and *ppepf1* mutants may improve our insight into the role of these genes in stomatal development.

Our experiments demonstrated that for both EPF1 and TMM conservation of function extends across the evolutionary distance separating bryophytes and angiosperms, with expression of *PpEPF1* and *PpTMM* coding sequences leading to a partial rescue of the mutant phenotype in the relevant *Arabidopsis* genetic backgrounds. Interestingly, overexpression of *PpEPF1* in the *atepf1* background was sufficient to restore stomatal number to near wild-type level whereas it was less able to rescue the related *atepf2* mutant phenotype. *AtEPF1* has been implicated in the spacing patterning of stomata whereas *AtEPF2* is thought to be more important for the earlier asymmetric divisions required for angiosperm stomatal initiation (Hara et al., 2007, 2009; Hunt and Gray, 2009). Our data therefore support the idea of an ancient role for an EPF peptide ligand in stomatal patterning, with the evolution of the angiosperm EPF gene family being linked to acquisition of asymmetrically dividing cells in stomatal development (Hara et al., 2009; Hunt and Gray, 2009). In *Arabidopsis*, the EPF(L) gene family appears to have expanded over evolutionary time so that particular combinations of different ligands and receptors function in different organs. This divergence of EPF function linked to increased plant complexity is supported by the observed inability of the *Arabidopsis* *STOMAGEN* sequence to alter stomatal patterning in *P. patens*. The acquisition of such novel regulators of stomata formation may reflect an evolutionary trend to more complex developmental systems, enabling a flexible control of stomatal pattern to allow plants to adapt organs to specific environments (Abrash and Bergmann, 2010; Hronková et al., 2015; Hunt and Gray, 2009; Rychel et al., 2010; Shpak et al., 2005; Takata et al., 2013).

In conclusion, our results establish that the members of an EPF/TMM/ERECTA ligand-receptor system are conserved between bryophytes and angiosperms, both in terms of the presence and expression of the relevant genes and in the functional conservation of their role in stomatal patterning. Our data do not provide information on the conservation (or otherwise) of the molecular interactions between the components of the EPF/TMM/ERECTA module (which have only recently become well-described in the more highly studied *Arabidopsis* system; Lee et al., 2015, 2012) and this represents an area for future investigation. Finally, the acquisition of stomata is recognised as being of fundamental importance in the evolution of land plants (Beerling, 2007; Berry et al., 2010) and our data strongly support the proposition that the genetic system regulating stomatal patterning was recruited at an extremely early stage of land plant evolution, supporting the idea that extant stomata are of monophyletic origin (Beerling, 2007; Edwards et al., 1998).

## MATERIALS AND METHODS

### Plant materials and growth conditions

*Physcomitrella patens* subspecies patens (Hedwig) strain ‘Gransden’ protonemal tissue and gametophores were grown at 25°C with continuous light [photosynthetically active radiation (PAR) 140 μmol m^−2^ s^−1^] in a Sanyo MLR-350 cabinet for transformations and genotyping. For stomatal analyses, *P. patens* was grown on sterile peat pellets under sporulating conditions (Chater et al., 2011). *Arabidopsis thaliana* seeds were surface sterilised, stratified and grown on M3 Levington compost in Conviron growth cabinets at 10 h 22°C/14 h 16-18°C light/dark cycle, 70% relative humidity, PAR 120 µmol m^−2^ s^−1^.

### Bioinformatic analysis

Sequences were aligned using the MUSCLE (Edgar, 2004) alignment tool on MEGA6 (Tamura et al., 2013). The evolutionary history was inferred using the neighbour-joining method (Saitou and Nei, 1987) on MEGA6 (Tamura et al., 2013). The optimal trees with the sum of branch length 13.422 (EPF), 9.345 (TMM) or 20.7487 (ERECTA) are shown. The percentage of replicate trees in which the associated taxa clustered together in the bootstrap test (1000 replicates) are shown next to the branches (Felsenstein, 1985). The tree is drawn to scale, with branch lengths in the same units as those of the evolutionary distances used to infer the phylogenetic tree. The evolutionary distances were computed using the Poisson correction method (Zuckerkandl and Pauling, 1965) and are in the units of the number of amino acid substitutions per site. For EPF(L) representatives, the analysis involved 79 amino acid sequences. All positions containing gaps and missing data were eliminated. There were a total of 33 positions in the final dataset. For TMM, the analysis involved 31 amino acid sequences. All positions containing gaps and missing data were eliminated. There were a total of 185 positions in the final dataset. For ERECTA, the analysis involved 82 amino acid sequences. All positions containing gaps and missing data were eliminated. There were a total of 24 positions in the final dataset. See Table S1 for accession numbers not indicated in the trees.

### *P. patens* gene manipulation and expression analysis

The 5′ and 3′ flanking regions of targeted genes were amplified from *P. patens* genomic DNA and inserted into plasmids by conventional cloning using primers detailed in Table S2. Resulting plasmids were used as PCR templates to amplify knockout constructs. *PpEPF1* was blunt-end ligated into *Eco*RV-digested pKS-Eco, then *Bso*BI digested, and a hygromycin selection cassette (obtained from pMBLH6bI) blunt-end ligated between 5′ and 3′ flanking regions to produce the *Ppepf* knockout construct. The *pptmm* and *pperecta1* knockout constructs were both created by blunt-end ligating 5′ flanking sequences into *Ecl*136II-digested pMBL5DLdelSN (a pMBL5 derivative) containing the NPTII cassette. Resulting plasmids were digested with *Eco*RV and 3′ flanking sequences inserted via blunt-end ligation. To target the *PpEPF1* locus in the *pptmm-1* background, the *Ppepf* knockout construct was used. To target the *PpERECTA1* locus in the *pptmm-1* background, the NPTII cassette in the *pperecta* construct was replaced with an HPH cassette at *Kpn*I and *Nsi*I sites to produce a hygromycin-selective *pperecta* knockout construct.

To target overexpression constructs of *PpEPF1*, *PpTMM* and *AtSTOMAGEN* to the neutral 108 locus, genes minus their ATG codon were amplified from *P. patens* cDNA (*PpEPF1*), genomic DNA (*PpTMM*) or *Arabidopsis* cDNA (*AtSTOMAGEN*). They were blunt-end ligated into *Nco*I-digested pACT-nos1, which contains the rice Actin-1 promoter and adjoining 5′ UTR (Horstmann et al., 2004; McElroy et al., 1990). pACT1-nos fused genes were PCR amplified, digested with *Kpn*I, and ligated into *Kpn*I/*Sma*I-digested pMBL5DL108 (Wallace et al., 2015).

Gene targeting and polyethylene glycol-mediated transformation of *P. patens* was performed using PCR-derived templates (Kamisugi et al., 2005; Schaefer et al., 1991). Confirmation of integration at target site was performed by genomic PCR analysis (Fig. S3). Briefly, for each independent line PCR was performed targeting a fragment spanning the 5′ genomic sequence to the transgene resistance cassette (Fig. S3B,D,F,H,J) or the 3′ genomic sequence to the transgene resistance cassette (Fig. S3C,E,G,I,K). For each gene knockout, either two or three independent transgenic lines were generated and analysed, with the exception of *pperecta1* and *pptmm-erecta1* for which only one line was obtained showing correct gene targeting and no expressed transcript. Expression of transgenes and absence of expression of targeted knockout genes was determined by RT-PCR using single-stranded cDNA generated from extracted RNA by M-MLV Reverse Transcriptase (Fisher Scientific). RNA was extracted using Spectrum Plant Total RNA Kit (Sigma). For expression analysis in *ppepf1* and *pptmm* lines, 120 developing sporophytes per line were harvested and used to extract RNA. For other RT-PCR analyses, gametophyte-sporophyte mix samples were collected for each line, which contained 25 gametophores and ∼15 developing sporophytes. For quantitative RT-PCR analysis of *PpERECTA1* transcript, relative expression was compared between RNA extracted from protonemal versus pooled sporophyte tissue (∼300 capsules per replicate: 100 immature, 100 mid-sized and 100 fully expanded sporophytes) in triplicated experiments. RNA integrity was verified by electrophoresis and NanoDrop ND-8000 (Fisher Scientific) and 1 µg RNA used in reactions alongside three control ‘housekeeping’ transcripts (Le Bail et al., 2013; Wolf et al., 2010), according to Luna et al. (2014) with slight modifications. Transcript abundance was assayed using Rotor-Gene SYBR Green PCR Kit and a Corbett Rotor Gene 6000 (Qiagen).

### *Arabidopsis thaliana* gene manipulation

For complementation experiments, *AtEPF2*pro and *AtEPF1*pro gene promoter sequences (Hunt and Gray, 2009) were amplified and ligated into *Kpn*I-digested pMDC99. Polished *Asc*I-digested *pMDC99::AtEPF1pro* was ligated with the *PpEPF1* gene product. *Asc*I/*Pac*I-digested *pMDC99::AtEPF2pro* was ligated with the *Asc*I-PpEPF1-*Pac*I product to produce the *AtEPF2::PpEPF1* fusion. For overexpression of *PpEPF1* in *Arabidopsis*, cDNA was amplified and inserted into *pENTR/D-TOPO* downstream of the 35S promoter of *pCTAPi* (Rohila et al., 2004) using LR Clonase. *AtTMMpro::PpTMM* fusions were constructed by ligating the *Asc*I-PpTMM-*Asc*I PCR product with *Asc*I-digested *pENTR::AtTMMpro* to produce *pENTR::AtTMMpro::PpTMM*. The promoter gene construct was then transferred to the HGW destination vector (Karimi et al., 2002) using LR clonase (Invitrogen). *Arabidopsis* wild type and mutants *epf1-1*, *epf2-2* and *tmm-1* in *Col-0* background (Hunt et al., 2010; Yang and Sack, 1995) were transformed using *Agrobacterium*-mediated floral dip (Clough and Bent, 1998). Transformants were selected and transgene insertion and expression verified by PCR and RT-PCR.

### Plant phenotyping

Fully expanded (orange to brown coloured) spore capsules were fixed in modified Carnoy's solution (2:1 ethanol: glacial acetic acid) 6 to 7 weeks after fertilisation by flooding. Capsules of a similar size were dissected to remove associated spores, mounted between a bridge of cover slides in distilled H_2_O and stomata imaged with an Olympus BX-51 microscope fitted with an Olympus DP71 camera and Olympus U-RFL-T-200 UV lamp equipped with an LP 400 nm emission filter. Multiple fields of view were stacked and colour corrected using ImageJ. Min Intensity (bright-field) or Max Intensity (fluorescence) settings were used to compile flattened images.

Fully expanded *Arabidopsis* leaves were collected 7 to 8 weeks after germination, abaxial epidermal impressions produced and stomatal densities taken from two to three fields of view per leaf (Hunt and Gray, 2009). Pedicels were collected from 14-week-old plants, fixed and cleared in modified Carnoy's solution, dissected longitudinally, rinsed in 0.5% diphenylboric acid-2-aminoethyl ester (DPBA) (Sigma-Aldrich) and 0.1% Triton X-100 (v/v) for 30 s, then mounted as above. Images were collected using bright-field on an Olympus BX-51 microscope with accompanying 400 nm fluorescence (pE-2 UV, CoolLED, Andover, UK) and 455 nm emission filter to capture fluorescence and stacked using ImageJ. Stomata were counted in areas of 180.26×262.56 µm approximately 300 µm from where pedicels were excised at the base, halfway up the stem and 150 µm from the tip. T2 and homozygous T3 plants were phenotyped. Statistical tests were performed using Graphpad Prism6 and graphs were produced using SigmaPlot version 13 (Systat Software).

## References

[DEV135038C1] Abrash E. B. and Bergmann D. C. (2010). Regional specification of stomatal production by the putative ligand CHALLAH. *Development* 137, 447-455. 10.1242/dev.040931 20056678

[DEV135038C2] Abrash E. B., Davies K. A. and Bergmann D. C. (2011). Generation of signaling specificity in arabidopsis by spatially restricted buffering of ligand-receptor interactions. *Plant Cell* 23, 2864-2879. 10.1105/tpc.111.086637 21862708 PMC3180797

[DEV135038C3] Beerling D. J. (2007). *The Emerald Planet: How Plants Changed Earth's History*. Oxford: Oxford University Press.

[DEV135038C4] Berry J. A., Beerling D. J. and Franks P. J. (2010). Stomata: key players in the earth system, past and present. *Curr. Opin. Plant Biol.* 13, 232-249. 10.1016/j.pbi.2010.04.013 20552724

[DEV135038C5] Bhave N. S., Veley K. M., Nadeau J. A., Lucas J. R., Bhave S. L. and Sack F. D. (2009). TOO MANY MOUTHS promotes cell fate progression in stomatal development of Arabidopsis stems. *Planta* 229, 357-367. 10.1007/s00425-008-0835-9 18979118

[DEV135038C5a] Chater C., Kamisugi Y., Movahedi M., Fleming A., Cuming A. C., Gray J. E. and Beerling D. J. (2011). Regulatory mechanism controlling stomatal behaviour conserved across 400 million years of land plant evolution. *Curr. Biol.* 21, 1025-1029. 10.1016/j.cub.2011.04.032 21658944

[DEV135038C6] Chater C., Gray J. E. and Beerling D. J. (2013). Early evolutionary acquisition of stomatal control and development gene signalling networks. *Curr. Opin. Plant Biol.* 16, 638-646. 10.1016/j.pbi.2013.06.013 23871687

[DEV135038C7] Chater C., Peng K., Movahedi M., Dunn J. A., Walker H. J., Liang Y.-K., McLachlan D. H., Casson S., Isner J. C., Wilson I. et al. (2015). Elevated CO2-induced responses in stomata require ABA and ABA signaling. *Curr. Biol.* 25, 2709-2716. 10.1016/j.cub.2015.09.013 26455301 PMC4612465

[DEV135038C8] Clough S. J. and Bent A. F. (1998). Floral dip: a simplified method for Agrobacterium-mediated transformation of Arabidopsis thaliana. *Plant J.* 16, 735-743. 10.1046/j.1365-313x.1998.00343.x 10069079

[DEV135038C8a] Edgar R. C. (2004). MUSCLE: multiple sequence alignment with high accuracy and high throughput. *Nucleic Acids Res.* 19, 1792-1797. 10.1093/nar/gkh340 15034147 PMC390337

[DEV135038C9] Edwards D., Kerp H. and Hass H. (1998). Stomata in early land plants: an anatomical and ecophysiological approach. *J. Exp. Bot.* 49, 255-278. 10.1093/jxb/49.Special_Issue.255

[DEV135038C10] Engineer C. B., Ghassemian M., Anderson J. C., Peck S. C., Hu H. H. and Schroeder J. I. (2014). Carbonic anhydrases, EPF2 and a novel protease mediate CO2 control of stomatal development. *Nature* 513, 246-250. 10.1038/nature13452 25043023 PMC4274335

[DEV135038C11] Felsenstein J. (1985). Confidence limits on phylogenies: an approach using the bootstrap. *Evolution* 39, 783-791. 10.2307/2408678 28561359

[DEV135038C12] Geisler M., Yang M. and Sack F. D. (1998). Divergent regulation of stomatal initiation and patterning in organ and suborgan regions of the Arabidopsis mutants too many mouths and four lips. *Planta* 205, 522-530. 10.1007/s004250050351 9684356

[DEV135038C13] Goodstein D. M., Shu S., Howson R., Neupane R., Hayes R. D., Fazo J., Mitros T., Dirks W., Hellsten U., Putnam N. et al. (2012). Phytozome: a comparative platform for green plant genomics. *Nucleic Acids Res.* 40, D1178-D1186. 10.1093/nar/gkr944 22110026 PMC3245001

[DEV135038C14] Hara K., Kajita R., Torii K. U., Bergmann D. C. and Kakimoto T. (2007). The secretory peptide gene EPF1 enforces the stomatal one-cell-spacing rule. *Genes Dev.* 21, 1720-1725. 10.1101/gad.1550707 17639078 PMC1920166

[DEV135038C15] Hara K., Yokoo T., Kajita R., Onishi T., Yahata S., Peterson K. M., Torii K. U. and Kakimoto T. (2009). Epidermal cell density is autoregulated via a secretory peptide, EPIDERMAL PATTERNING FACTOR 2 in arabidopsis leaves. *Plant Cell Physiol.* 50, 1019-1031. 10.1093/pcp/pcp068 19435754

[DEV135038C16] Horstmann V., Huether C. M., Jost W., Reski R. and Decker E. L. (2004). Quantitative promoter analysis in Physcomitrella patens: a set of plant vectors activating gene expression within three orders of magnitude. *BMC Biotechnol.* 4, 13-13 10.1186/1472-6750-4-13 PMC490084 15239842

[DEV135038C17] Hronková M., Wiesnerová D., Šimková M., Skůpa P., Dewitte W., Vráblová M., Zažímalová E. and Šantrůček J. (2015). Light-induced STOMAGEN-mediated stomatal development in Arabidopsis leaves. *J. Exp. Bot.* 66, 4621-4630. 10.1093/jxb/erv233 26002974

[DEV135038C18] Hunt L. and Gray J. E. (2009). The signaling peptide EPF2 controls asymmetric cell divisions during stomatal development. *Curr. Biol.* 19, 864-869. 10.1016/j.cub.2009.03.069 19398336

[DEV135038C19] Hunt L., Bailey K. J. and Gray J. E. (2010). The signalling peptide EPFL9 is a positive regulator of stomatal development. *New Phytol.* 186, 609-614. 10.1111/j.1469-8137.2010.03200.x 20149115

[DEV135038C20] Kamisugi Y., Cuming A. C. and Cove D. J. (2005). Parameters determining the efficiency of gene targeting in the moss Physcomitrella patens. *Nucleic Acids Res.* 33, e173 10.1093/nar/gni172 16282584 PMC1283530

[DEV135038C21] Karimi M., Inzé D. and Depicker A. (2002). GATEWAY™ vectors for Agrobacterium-mediated plant transformation. *Trends Plant Sci.* 7, 193-195. 10.1016/S1360-1385(02)02251-3 11992820

[DEV135038C22] Le Bail A., Scholz S. and Kost B. (2013). Evaluation of reference genes for RT qPCR analyses of structure-specific and hormone regulated gene expression in physcomitrella patens gametophytes. *PLoS ONE* 8, e70998 10.1371/journal.pone.0070998 23951063 PMC3739808

[DEV135038C23] Lee J. S., Kuroha T., Hnilova M., Khatayevich D., Kanaoka M. M., McAbee J. M., Sarikaya M., Tamerler C. and Torii K. U. (2012). Direct interaction of ligand-receptor pairs specifying stomatal patterning. *Genes Dev.* 26, 126-136. 10.1101/gad.179895.111 22241782 PMC3273837

[DEV135038C24] Lee J. S., Hnilova M., Maes M., Lin Y.-C. L., Putarjunan A., Han S.-K., Avila J. and Torii K. U. (2015). Competitive binding of antagonistic peptides fine-tunes stomatal patterning. *Nature* 522, 439-443. 10.1038/nature14561 26083750 PMC4532310

[DEV135038C25] Luna E., van Hulten M., Zhang Y. H., Berkowitz O., López A., Pétriacq P., Sellwood M. A., Chen B. N., Burrell M., van de Meene A. et al. (2014). Plant perception of beta-aminobutyric acid is mediated by an aspartyl-tRNA synthetase. *Nat. Chem. Biol.* 10, 450-456. 10.1038/nchembio.1520 24776930 PMC4028204

[DEV135038C26] MacAlister C. A. and Bergmann D. C. (2011). Sequence and function of basic helix-loop-helix proteins required for stomatal development in Arabidopsis are deeply conserved in land plants. *Evol. Dev.* 13, 182-192. 10.1111/j.1525-142X.2011.00468.x 21410874 PMC3139685

[DEV135038C27] MacAlister C. A., Ohashi-Ito K. and Bergmann D. C. (2007). Transcription factor control of asymmetric cell divisions that establish the stomatal lineage. *Nature* 445, 537-540. 10.1038/nature05491 17183265

[DEV135038C28] McElroy D., Zhang W., Cao J. and Wu R. (1990). Isolation of an efficient actin promoter for use in rice transformation. *Plant Cell* 2, 163-171. 10.1105/tpc.2.2.163 2136633 PMC159873

[DEV135038C29] O'Donoghue M.-T., Chater C., Wallace S., Gray J. E., Beerling D. J. and Fleming A. J. (2013). Genome-wide transcriptomic analysis of the sporophyte of the moss Physcomitrella patens. *J. Exp. Bot.* 64, 3567-3581. 10.1093/jxb/ert190 23888066 PMC3745722

[DEV135038C30] Ohki S., Takeuchi M. and Mori M. (2011). The NMR structure of stomagen reveals the basis of stomatal density regulation by plant peptide hormones. *Nat. Commun.* 2, 512 10.1038/ncomms1520 22027592

[DEV135038C31] Ortiz-Ramírez C., Hernandez-Coronado M., Thamm A., Catarino B., Wang M., Dolan L., Feijó J. A. and Becker J. D. (2016). A transcriptome atlas of *Physcomitrella patens* provides insights into the evolution and development of land plants. *Mol. Plant* 9, 205-220. 10.1016/j.molp.2015.12.002 26687813

[DEV135038C32] Peterson K. M., Rychel A. L. and Torii K. U. (2010). Out of the mouths of plants: the molecular basis of the evolution and diversity of stomatal development. *Plant Cell* 22, 296-306. 10.1105/tpc.109.072777 20179138 PMC2845417

[DEV135038C33] Pillitteri L. J. and Torii K. U. (2012). Mechanisms of stomatal development. In *Annual Review of Plant Biology*, Vol. 63 (ed. Merchant S. S.), pp. 591-614. Palo Alto: Annual Reviews. 10.1146/annurev-arplant-042811-105451 22404473

[DEV135038C34] Raven J. A. (2002). Selection pressures on stomatal evolution. *New Phytol.* 153, 371-386. 10.1046/j.0028-646X.2001.00334.x 33863217

[DEV135038C35] Rohila J. S., Chen M., Cerny R. and Fromm M. E. (2004). Improved tandem affinity purification tag and methods for isolation of protein heterocomplexes from plants. *Plant J.* 38, 172-181. 10.1111/j.1365-313X.2004.02031.x 15053770

[DEV135038C36] Ruszala E. M., Beerling D. J., Franks P. J., Chater C., Casson S. A., Gray J. E. and Hetherington A. M. (2011). Land plants acquired active stomatal control early in their evolutionary history. *Curr. Biol.* 21, 1030-1035. 10.1016/j.cub.2011.04.044 21658945

[DEV135038C37] Rychel A. L., Peterson K. M. and Torii K. U. (2010). Plant twitter: ligands under 140 amino acids enforcing stomatal patterning. *J. Plant Res.* 123, 275-280. 10.1007/s10265-010-0330-9 20336477

[DEV135038C38] Saitou N. and Nei M. (1987). The neighbor-joining method: a new method for reconstructing phylogenetic trees. *Mol. Biol. Evol.* 4, 406-425. 3447015 10.1093/oxfordjournals.molbev.a040454

[DEV135038C39] Schaefer D., Zryd J.-P., Knight C. D. and Cove D. J. (1991). Stable transformation of the moss Physcomitrella patens. *Mol. Gen. Genet.* 226, 418-424. 10.1007/BF00260654 2038304

[DEV135038C40] Shpak E. D., McAbee J. M., Pillitteri L. J. and Torii K. U. (2005). Stomatal patterning and differentiation by synergistic interactions of receptor kinases. *Science* 309, 290-293. 10.1126/science.1109710 16002616

[DEV135038C41] Simmons A. R. and Bergmann D. C. (2016). Transcriptional control of cell fate in the stomatal lineage. *Curr. Opin. Plant Biol.* 29, 1-8. 10.1016/j.pbi.2015.09.008 26550955 PMC4753106

[DEV135038C41a] Steeves T. A. and Sussex I. M. (1989). *Patterns in Plant Development*, 2nd edn. Cambridge: Cambridge University Press.

[DEV135038C42] Sugano S. S., Shimada T., Imai Y., Okawa K., Tamai A., Mori M. and Hara-Nishimura I. (2010). Stomagen positively regulates stomatal density in Arabidopsis. *Nature* 463, 241-244. 10.1038/nature08682 20010603

[DEV135038C43] Takata N., Yokota K., Ohki S., Mori M., Taniguchi T. and Kurita M. (2013). Evolutionary relationship and structural characterization of the EPF/EPFL gene family. *PLoS ONE* 8, e65183 10.1371/journal.pone.0065183 23755192 PMC3670920

[DEV135038C44] Tamura K., Stecher G., Peterson D., Filipski A. and Kumar S. (2013). MEGA6: molecular evolutionary genetics analysis version 6.0. *Mol. Biol. Evol.* 30, 2725-2729. 10.1093/molbev/mst197 24132122 PMC3840312

[DEV135038C45] Torii K. (2012). Mix-and-match: ligand–receptor pairs in stomatal development and beyond. *Trends Plant Sci.* 17, 711-719. 10.1016/j.tplants.2012.06.013 22819466

[DEV135038C46] Torii K. U. (2015). Stomatal differentiation: the beginning and the end. *Curr. Opin. Plant Biol.* 28, 16-22. 10.1016/j.pbi.2015.08.005 26344486

[DEV135038C47] Vatén A. and Bergmann D. C. (2012). Mechanisms of stomatal development: an evolutionary view. *Evodevo* 3, 11 10.1186/2041-9139-3-11 22691547 PMC3390899

[DEV135038C48] Villagarcia H., Morin A.-C., Shpak E. D. and Khodakovskaya M. V. (2012). Modification of tomato growth by expression of truncated ERECTA protein from Arabidopsis thaliana. *J. Exp. Bot.* 63, 6493-6504. 10.1093/jxb/ers305 23096000

[DEV135038C49] Wallace S., Chater C. C., Kamisugi Y., Cuming A. C., Wellman C. H., Beerling D. J. and Fleming A. J. (2015). Conservation of Male Sterility 2 function during spore and pollen wall development supports an evolutionarily early recruitment of a core component in the sporopollenin biosynthetic pathway. *New Phytol.* 205, 390-401. 10.1111/nph.13012 25195943

[DEV135038C50] Winter D., Vinegar B., Nahal H., Ammar R., Wilson G. V. and Provart N. J. (2007). An “electronic fluorescent pictograph” browser for exploring and analyzing large-scale biological data sets. *PLoS ONE* 2, e718 10.1371/journal.pone.0000718 17684564 PMC1934936

[DEV135038C51] Wolf L., Rizzini L., Stracke R., Ulm R. and Rensing S. A. (2010). The molecular and physiological responses of physcomitrella patens to ultraviolet-B radiation. *Plant Physiol.* 153, 1123-1134. 10.1104/pp.110.154658 20427465 PMC2899899

[DEV135038C52] Yang M. and Sack F. D. (1995). The too many mouths and four lips mutations affect stomatal production in arabidopsis. *Plant Cell* 7, 2227-2239. 10.1105/tpc.7.12.2227 11536724 PMC161075

[DEV135038C53] Zuckerkandl E. and Pauling L. (1965). Molecules as documents of evolutionary history. *J. Theor. Biol.* 8, 357-366. 10.1016/0022-5193(65)90083-4 5876245

